# Environmental regulations, capacity utilization, and high-quality development of manufacturing: an analysis based on Chinese provincial panel data

**DOI:** 10.1038/s41598-021-98787-y

**Published:** 2021-10-01

**Authors:** Hongyang Wang, Baizhou Li

**Affiliations:** School of Economics and Management, Harbin Engineering University, Harbin, 150001 China

**Keywords:** Environmental social sciences, Environmental economics, Environmental impact, Sustainability

## Abstract

The high-quality development of the manufacturing industry is an important strategic task for Chinese economic development. The rapid development of the manufacturing industry is also accompanied by problems such as overcapacity and environmental pollution. This paper analyzes the impact of capacity utilization on the high-quality development of manufacturing and establishes a nonlinear threshold regression model on this basis, and studies and analyzes environmental regulations as a threshold variable under the influence of capacity utilization rate on the high-quality development of the manufacturing industry. The research results show that: capacity utilization, profitability, foreign direct investment, and government participation all have a significant positive impact on the high-quality development of the manufacturing industry; environmental regulations have a significant negative impact on the high-quality development of the manufacturing industry. And in the model of the effect of capacity utilization on the high-quality development of the manufacturing industry, environmental regulation has a single threshold effect. With the increase in the intensity of environmental regulation, the coefficient and significance of the effect of capacity utilization on the high-quality development of the manufacturing industry have changed. Finally, this article puts forward corresponding policies and suggestions based on the results of data analysis.

## Introduction

Manufacturing is an important way to promote economic growth. From the "Reindustrialization" development strategy put forward by the United States, Germany, Britain, France, and the European Union and made in China 2025 issued by China, the importance of manufacturing has been re-recognized by countries all over the world. Manufacturing is an important driving force for China's economic growth^1^. The 2018 Central Economic Work Conference put the promotion of high-quality development of manufacturing at the top of its key tasks, and the 2019 government work report once again emphasized the promotion of high-quality development of manufacturing^2^. Since China’s reform and opening up, while the development speed and overall scale of China’s manufacturing industry have made remarkable achievements, they are also facing problems such as overcapacity, environmental pollution, and energy consumption that threaten the sustainable development of China’s economy. To a certain extent, it has delayed the pace of China's economic development towards high-quality development^3^. The high-quality development of the manufacturing industry is the only choice for China's manufacturing industry to turn from "large" to "strong"^2^. Environmental pollution, ecological destruction, and increasingly scarce resources and energy are serious challenges faced by all countries in the world. The development of green and clean production and the realization of sustainable development have become the consensus of the international community. The “Guiding Opinions on Resolving Serious Overcapacity Contradictions” issued by the State Council in 2013 clearly stated that strengthening environmental access and strengthening environmental law enforcement are effective measures to resolve overcapacity. Environmental standards for industries with overcapacity must be gradually improved to guide the green development of industries^5^. In the "2016 Environmental Performance Index Report" released by Yale University in 2016, China's Environmental Performance Index (EPI) ranked second, and environmental issues have attracted great attention from the general public and governments at all levels^4^. How to achieve the win-win goal of improving environmental quality and increasing industrial capacity utilization has gradually become a hot topic in the academic circles under the background of high-quality economic development in China in the new era.

Overcapacity is an important issue of general concern to the international community after the international financial crisis. Overcapacity has seriously hindered the sustainable development of China's economy. Overcapacity refers to the situation where the production capacity formed in advance exceeds the equilibrium quantity requirement, which leads to the idle situation of production factors. The "Guidelines of the State Council on Solving the Contradictions of Serious Overcapacity" promulgated by the government in 2013 have had a huge external impact on the traditionally competitive manufacturing market^6–8^. In terms of the inspection of capacity utilization and the judgment of overcapacity, according to the analysis results published by the International Monetary Fund (IMF2012), China's overall capacity utilization rate dropped from around 85% at the turn of the century to 60% at 2011. In addition, according to the analysis results of Chinese scholars, the average capacity utilization rate of China's industry from 2001 to 2011 was 69.3%. Taking 2008 as the demarcation point, it showed an upward trend before, and then basically showed a fluctuating downward trend^9^. From 2011 to 2014, the average utilization rate of China's manufacturing capacity was only 70% to 75%. The capacity utilization rate of most industries was lower than the internationally recognized normal level of 79–82%. Generally speaking, more than 85% indicates insufficient production capacity. Below 75% indicates serious overcapacity. Overcapacity has now become the source of prominent contradictions and many economic problems in the process of structural adjustment of China’s manufacturing industry. Low-level capacity utilization has led to problems such as lower profits, underutilization, business closures, and layoffs. On the other hand, excess capacity will lead to Vicious market competition, waste of resources, and environmental pollution. These negative effects will affect the industrial upgrading by reducing the investment in technological innovation of enterprises, reducing the efficiency of resource allocation in the industry, and insufficient demand for market products, which in turn will affect the high-quality development of the manufacturing industry^10^.

Sustainable development has become a consensus of mankind. Energy production drives the development of all walks of life. Industrialization in different countries in the world requires more energy and corresponding upstream and downstream processes, which may lead to a significant increase in environmental hazards^11^. China’s economic development is facing the dual pressures of the structural transformation of the manufacturing industry and the constraints of the ecological environment. The rapid economic development has also brought tremendous pressure to the environment. For the sustainable development of the manufacturing industry, reducing the pollution emissions of the manufacturing industry is very important, and the use of environmental regulation to control pollution has become an inevitable trend^12,13^. China's manufacturing industry is still at the middle and low end of the industrial value chain, and excess production capacity should be eliminated as much as possible to reduce the impact on the environment. The main goal of environmental issues is to make environmental sustainability the highest priority and to ensure that environmental issues are incorporated into their strategic considerations^3^, the manufacturing industry is the main body of energy consumption and pollution emissions. The implementation of environmental regulations is conducive to the protection and restoration of China’s natural and living environments, but it also inevitably affects the daily operation of other industries including manufacturing^14,15^. Appropriate environmental regulation can promote enterprise performance. Environmental regulations will force the technological innovation of the manufacturing industry, and technological innovation will promote the high-quality development of the manufacturing industry by accelerating the transformation of the development mode, optimizing the industrial structure of the manufacturing industry, and improving the international competitiveness of the manufacturing industry^2^. How will overcapacity affect the high-quality development of manufacturing? Can environmental regulations promote the high-quality development of manufacturing? Is there a non-linear effect? Based on the panel data of 30 provinces and cities in China from 2005 to 2015, this article studies and analyzes the impact of capacity utilization on the high-quality development of the manufacturing industry from the linear and nonlinear perspectives, and establishes a nonlinear threshold regression model to study and analyze the impact of capacity utilization on the high-quality development of manufacturing industry with environmental regulations as the threshold variable. To provide theoretical support for the successful implementation of high-quality development of the manufacturing industry.

## Literature review and research hypothesis

### The impact of capacity utilization on high-quality development of manufacturing industry

Capacity utilization (CU) is a comprehensive method to measure the degree of overcapacity in an industry. CU of an industry is equal to the ratio of actual output to industrial potential output^16^, in western countries, overcapacity is a relative concept and an inevitable result of market competition. In a certain time, industries with excess capacity will evolve into balanced industries and become industries with market survival of the fittest and economic environment changes^16^. From an economic perspective, CU is measured by the ratio of actual economic output to potential economic output^17^. With the cyclical fluctuations of the economy, overcapacity occurs from time to time. Generally speaking, when a country's domestic supply exceeds effective demand, there will be overcapacity^7^. Yang's research shows that the CU level of China's manufacturing industry had improved from 2007 to 2010, which means that China's manufacturing industry has expanded production and is closer to capacity during this period. The average CU value of the light industry was higher than that of heavy industry, and the two light industries had extremely high CU values, which shows that the light industry has a serious overcapacity problem^3^. Wang conducted an empirical investigation on green CU in 28 provinces of the Chinese mainland from 2010 to 2015. The results show that: first, China's high-tech industry shows a good overall performance in green Cu, with the western region having the highest green CU, followed by the central and northeast regions, and the eastern region has the lowest green CU^18^. Zhou's research shows that local government intervention has a serious negative impact on regional industrial utilization, and the inhibition degree of factor market segmentation on regional industrial utilization is higher than that of commodity market segmentation^19^. The report of the 19th National Congress of the Communist Party of China pointed out that to build a modern economic system, we must adhere to the principle of quality first and benefit first, take the supply-side structural reform as the mainline, and promote the quality, efficiency and power changes of economic development. The quality development of the manufacturing industry should start from quality, efficiency, and power. Optimize the industrial structure, enhance the innovation ability of the main body, strengthen the element support, and boost the high-quality development of the manufacturing industry^2^. High-quality development is based on the principle of quality first and benefit first. At present, most of the literature is studying the causes of overcapacity, while there are few studies on the influencing factors of high-quality development of the manufacturing industry. Industrial structure adjustment is an important part of the high-quality development of the manufacturing industry. From the perspective of economic benefits, scholars generally believe that overcapacity has become the root of prominent contradictions and many economic problems in the process of structural adjustment of China's manufacturing industry. Xiong constructed the evaluation index system of high-quality economic development and concluded that the capacity utilization rate has a significant role in promoting the high-quality economic development of China's regions, and the eastern region has the most serious impact on the high-quality economic development, while the western region has the least impact^20^. Lv's research shows that overcapacity and import volume will hurt enterprise income^21^. Zheng believes that overcapacity will hurt the economy. A low level of capacity utilization will lead to problems such as lower production costs and reduced management fees. Moreover, overcapacity will lead to vicious competition in the market, waste of resources, and environmental pollution. The resulting negative effects will affect the reduction of enterprise profits and cause foreign investors to withdraw their capital one after another. Therefore, it will affect enterprises' access to foreign technology, Therefore, the investment in technological innovation of enterprises will be reduced. If the technological innovation investment of enterprises is affected, the transformation of enterprises will be difficult, which will affect the upgrading of the manufacturing industry, and ultimately affect the high-quality development of the manufacturing industry^10,22^. Zheng analyzed the transmission mechanism of the impact of overcapacity on the upgrading of manufacturing industry from three dimensions of enterprise micro aspect, industry meso aspect, and national macro aspect, and came to the conclusion that: in terms of nationwide, the change of capacity utilization rate has a significant positive impact on the upgrading of manufacturing industry; in terms of different types of manufacturing industry, the impact of capacity utilization rate on the upgrading of light industry manufacturing industry is significant For different regions, the eastern region mainly depends on the improvement of the capacity utilization rate of heavy industry manufacturing industry to promote industrial upgrading, while the central and western regions mainly rely on the improvement of the capacity utilization rate of light industry manufacturing industry to promote industrial upgrading^10^.

#### Hypothesis H1

Under the condition that other conditions remain unchanged, capacity utilization has a positive impact on the high quality of the manufacturing industry.

### The role of environmental regulation in the impact of capacity utilization on the high-quality development of the manufacturing industry

Nowadays, most kinds of literature are studying the causes of overcapacity, and lack of research on capacity utilization from the perspective of environmental regulation. Facing the increasing attention to the current environmental problems, some scholars began to try to study the problem of overcapacity from the perspective of the ecological environment^23^. The rapid development of China's manufacturing industry also brings about the consumption of resources and the destruction of the environment. Nearly one-third of the global energy consumption and 36% of the carbon dioxide emissions are attributed to the manufacturing industry^24^. Environmental supervision refers to the situation that the government formulates environmental standards, formulates supervision and punishment means, and solves the negative externality problem of economic entity pollution, to realize the more harmonious development of resources, environment, and economy^25^. China's industrial economy has a large-scale and complex production structure. Therefore, improving the environmental and economic performance of industrial enterprises has become an important goal of national environmental governance^26^. Environmental regulation is an important method to control environmental pollution. Environmental regulation can effectively reduce environmental pollution, but also has an impact on capacity utilization. Many scholars have studied the relationship between environmental management and enterprise economic performance. Some enterprises believe that environmental management realizes the win-win of environmental protection and economic performance, but many enterprises believe that environmental management will reduce the income scale of enterprises^27^. Zhou's research shows that there is a negative correlation between environmental regulation and enterprise financial performance, and the government's environmental regulation policy has a restrictive effect on enterprise financial performance^28^. Most scholars believe that the environmental laws and regulations implemented by the government will promote the generation of innovative technologies and play a certain role in improving the capacity utilization rate of enterprises^29^. Some scholars have shown that environmental regulation has a significant impact on overcapacity and industrial structure adjustment. Higher environmental regulation will increase overcapacity, while moderate environmental regulation will optimize industrial structure^29^. Some scholars believe that environmental governance will directly increase the production costs of enterprises and reduce the opportunities for other investment projects, thus hindering the improvement of capacity utilization^1^. Kong believes that market incentive environmental regulation has more effectively promoted the improvement of industrial capacity utilization, and proposes to strengthen the positive role of market incentive environmental regulation, timely and moderately improve industrial capacity utilization, and gradually achieve the goal of high-quality industrial development^5^. Yu studies the impact of environmental regulation intensity on industrial capacity utilization and also studies the compliance cost effect and innovation offset effect of environmental regulation intensity on industrial capacity utilization. It is found that there are significant differences in environmental supervision intensity and industrial capacity utilization among provinces in China, and there is a trend of fluctuation but rising gradually. At the same time, the utilization rate of industrial capacity also has a significant spatial correlation, and the increase of environmental governance can significantly improve the utilization rate of industrial capacity. Environmental regulations can also improve industrial capacity utilization through the innovation offset effect, but the compliance cost effect of environmental regulation is not significant^25^. Zheng believes that when companies have difficulties in production and operation, they will turn to other industries to seek new profit growth points, which will play an important role in the adjustment of industrial structure, and changes in industrial structure will affect overcapacity. Through the market compulsory mechanism, environmental regulation will not only affect the overcapacity but also adjust the industrial structure^29^.

#### Hypothesis H2

Under the condition that other conditions remain unchanged, In the role model of capacity utilization on the high-quality development of the manufacturing industry, environmental regulation has a threshold effect.

**Figure 1:**
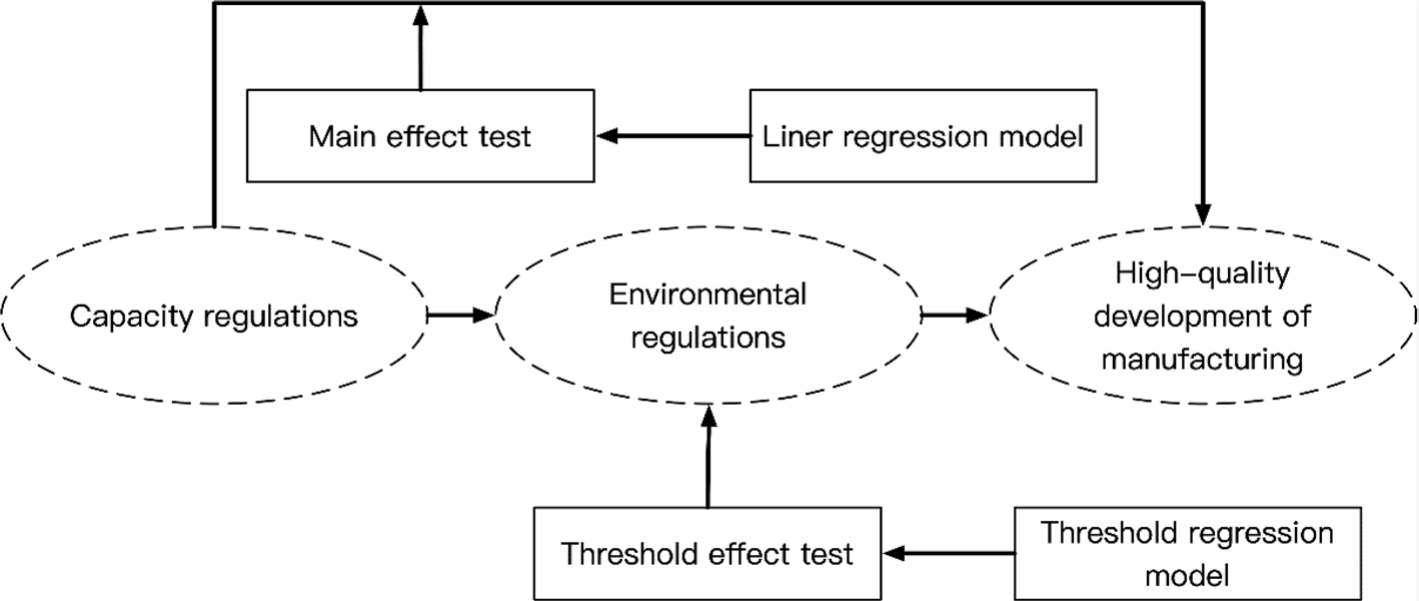
The conceptual framework.

The conceptual framework in this study is shown in Fig. 1.

## Variable selection and data source

### Variable selection

#### High-quality development of manufacturing industry (quality)

Referring to the index system used by Tang Hongxiang, this article measures the high-quality development of the manufacturing industry from the perspective of economic development quality of the manufacturing industry. As shown in Table 1. Select the development speed, development efficiency, and high-end industrial structure of the manufacturing industry to measure the high-quality development level of the manufacturing industry^30^. Specifically, the development speed of the manufacturing industry is measured by the growth rate of the manufacturing industry: first, use the output value of the next year of the manufacturing industry minus the output value of the previous year, and then use the obtained value divided by the output value of the previous year; The development benefit is measured by the profit rate of the main business: operating profit divided by the main business income. The high-end industrial structure is measured by the ratio of the output value of the high-end manufacturing industry divided by the output value of the manufacturing industry. The entropy method is used to calculate the weight of each indicator, and then the comprehensive value is obtained through calculation.

**Table 1:** Evaluation indicators for high-quality development of manufacturing industry.

	Index	Indicator meaning
Quality	Development speed	The growth rate of the manufacturing industry = (the output value of the next year of the manufacturing industry- the output value of the previous year)/the output value of the previous year
	Development efficiency	The profit rate of the main business = operating profit/the main business income
	The high-end industrial structure of the manufacturing industry	The high-end industrial structure of the manufacturing industry = the output value of the high-end manufacturing industry/output value of the manufacturing industry

The Organization for Economic Cooperation and Development (OECD) divides the manufacturing structure into four types of industries: low-end, mid-low-end, mid-high-end, and high-end industries according to technical levels. This paper refers to the classification method of Fu^31^, merges high-end and mid-to-high-end industries, and divides manufacturing into three categories. High-end technology industries include general equipment, special equipment, transportation, electrical machinery and equipment, communications, electronics, instrumentation and cultural office machinery, chemical and pharmaceutical industries; mid-end technology industries include petroleum processing, coking, and nuclear fuel processing industries, rubber, Plastics, non-metallic minerals, golden metal smelting, non-ferrous metal smelting, and metal products industries^31^.

**Figure 2:**
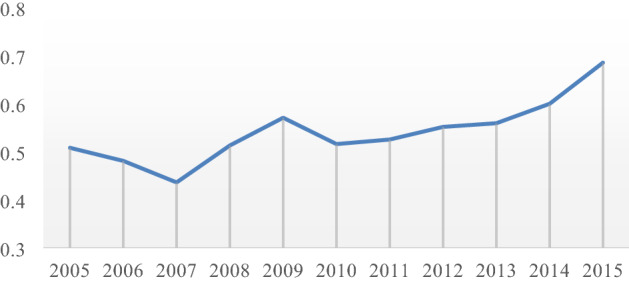
The overall development quality score of China's manufacturing industries.
  The overall development quality score of China's manufacturing industry is low, ranging from 0.43 to 0.68 in 2005–2015, and the lowest score index in 2007 was 0.435. Since 2010, the overall development quality of the manufacturing industry has gradually begun to grow slowly. It shows that the structural transformation and high-quality development of the manufacturing industry have become the mainstream consciousness in China.

Step 1: Standardization of indicators
  This article selects 11 years, 30 regions, and 3 indicators, ${X}_{\theta ij}$ is the value of the $j$-th index in the $i$-th area in year $\theta$ ($i=\mathrm{1,2}..,30; \; \; j=\mathrm{1,2},\dots ,11$). Since the measurement units of the various indicators are not uniform, before using them to calculate the comprehensive indicators, they must be standardized, that is, the absolute value of the indicator is converted into a relative value.
  Since the three indicators in this analysis are all positive indicators, we only analyze the positive indicators:
  1 $$\begin{array}{c}{x}_{\theta ij}^{\mathrm{^{\prime}}}=\frac{{x}_{\theta ij}-min\left\{{x}_{\theta 1j},\ldots ,{x}_{\theta 30j}\right\}}{max\left\{{x}_{\theta 1j},\ldots ,{x}_{\theta 30j}\right\}-min\left\{{x}_{\theta 1j},\ldots ,{x}_{\theta 30j}\right\}}\end{array}$$
Step 2: Determine the weight of the indicator
  Calculate the proportion of the $j$-th indicator in the $i$-th area in year $\theta$ of the indicator:
  2 $$\begin{array}{c}{y}_{\theta ij}=\frac{{x}_{\theta ij}^{\mathrm{^{\prime}}}}{{\sum }_{\theta }{\sum }_{i}{x}_{\theta ij}^{\mathrm{^{\prime}}}}\end{array}$$
Step 3: Calculate the entropy value of the ${\varvec{j}}$-th index
  3 $$\begin{array}{c}{e}_{j}=-k{\sum }_{\theta }{\sum }_{i}{y}_{ij}ln\left({y}_{\theta ij}\right)\end{array}$$
  4 $$\begin{array}{c}k>0,k=ln\left(m\right),m=3\end{array}$$
  Note: To avoid the situation where $k=ln\left(0\right)$ cannot be calculated, the displacement method is used here, the amount of displacement is + 0.00001, that is:
  When $${y}_{\theta ij}=0:$$
  5 $$\begin{array}{c}{e}_{j}=-k{\sum }_{\theta }{\sum }_{i}{y}_{ij}ln\left({y}_{\theta ij}+0.00001\right)\end{array}$$
Step 4: Calculate the information utility value of the ${\varvec{j}}$-th index
  6 $$\begin{array}{c}{g}_{j}=1-{e}_{j}\end{array}$$
  After the above calculations, calculate the average value of the manufacturing high-quality development scores of each province and city in each year and then plot the average value of the high-quality development index of the manufacturing industry in each year as shown in Fig. 2.

#### Capacity utilization (CU)

This paper uses the input-based bcc model in DEA to measure China's regional capacity utilization rate. The production function used in this paper includes one output—the total output value of manufacturing, and three inputs—fixed capital, labor, and intermediate inputs. The algorithm of the fixed capital stock refers to the algorithm of Zhang^32^, using the perpetual inventory method to estimate.

7 $$\begin{array}{c}{K}_{t}={K}_{t-1}\left(1-{\delta }_{t}\right)+{I}_{t}/{P}_{t}\end{array}$$

Specifically, ${K}_{t-1}$ and ${K}_{t}$ respectively represent the stock of fixed capital in period $t-1$ and the stock of fixed capital in period $t$, ${\delta }_{t}$ represents the depreciation rate in period $t$, ${I}_{t}$ represents the amount of new investment in period t, ${P}_{t}$ represents the price index of investment products; The number of employees in the secondary industry is selected as a measure of labor input. The annual average value of fixed assets is used as a measure of intermediate input^33^.

Refer to the algorithm of Shen^34^, this paper uses $Y$ to represent the capacity output, which means the ideal output of the overall decision-making unit under the condition of optimal technical efficiency. The fixed factor of production input is denoted as $F$, and the production capacity is denoted as $Y(F)$. Use $y$ to represent the actual output of the decision-making unit, the extent to which production capacity can be converted into actual output depends on the technical level $TE$ and the variable production factors of input $V$. Therefore, the actual output is expressed as:

8 $$\begin{array}{c}y=Y\left(F,V,TE\right)\end{array}$$

Use the technical efficiency measured by the DEA method to replace the technical level $TE$, therefore, the actual output can also be expressed as:

9 $$\begin{array}{c}y=TE\times Y\left(F,V\right)\end{array}$$

According to the foregoing description, the capacity utilization rate $CU$ is the ratio between the actual output and the production capacity, which is:

10 $$\begin{array}{c}CU=y/Y=TE\times Y\left(F,V\right)/Y\left(F\right)\end{array}$$

$Y(F,V)/Y(F)$ can be understood as the utilization of equipment under the constraints of variable input elements. The equipment utilization rate is expressed in $EU$, which is:

11 $$\begin{array}{c}EU=Y\left(F,V\right)/Y\left(F\right)\end{array}$$

For $Y(F,V), Y(F)$, we use a variable return to scale method DEA-BCC for measurement:

12 $$\begin{array}{c}{Y}^{*}=maxY\left(F,V\right)\end{array}$$

13 $$\begin{array}{c}s.t\sum_{i=1}^{N}{\lambda }_{i}{V}_{i}\le {V}_{0},\end{array}$$

14 $$\begin{array}{c}\sum_{i=1}^{N}{\lambda }_{i}{F}_{i}\le {F}_{0}\end{array}$$

15 $$\begin{array}{c}\sum_{i=1}^{N}{\lambda }_{i}{y}_{i}\ge {y}_{0}\end{array}$$

16 $$\begin{array}{c}\sum_{i=1}^{N}{\lambda }_{i}=1,{\lambda }_{i}\ge 0\end{array}$$

17 $$\begin{array}{c}\widehat{{Y}^{*}}=\max Y\left(F\right)\end{array}$$

18 $$\begin{array}{c}s.t\sum_{i=1}^{N}{\lambda }_{i}{F}_{i}\le {F}_{0},\sum_{i=1}^{N}{\lambda }_{i}{y}_{i}\ge {y}_{0}\sum_{i=1}^{N}{\lambda }_{i}=1,{\lambda }_{i}\ge 0\end{array}$$

${\lambda }_{i}$ is the weight vector, n is the number of production units, i represents each production unit, $\sum_{i=1}^{N}{\lambda }_{i}=1$ means that the return to scale is variable.

**Figure 3:**
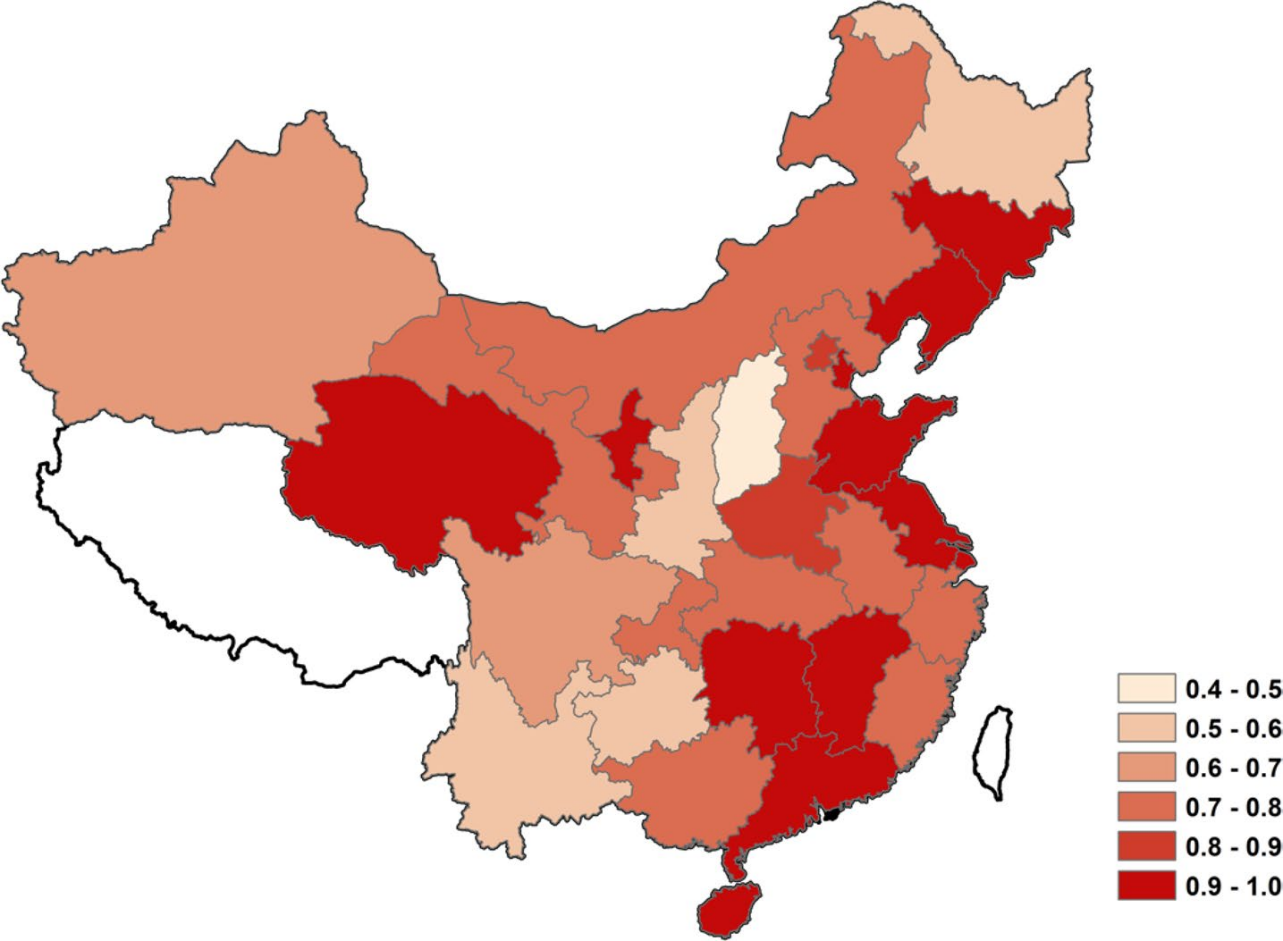
The average value of the capacity utilization rate of each region. The different colors represent the average value of the capacity utilization rate of each region, the color layer was created by the ArcGIS Pro 2.8. (https://pro.arcgis.com/en/pro-app/latest/get-started/download-arcgis-pro.htm).

After calculation, the capacity utilization rate of each region from 2005 to 2015 is obtained. Take the average value of the capacity utilization rate of each region and plot it as shown in Fig. 3:

It can be seen that the general capacity utilization rate in eastern China has a high score, mostly above 0.8. Most provinces in the remaining regions have low scores, and many regions are below 0.6. This shows that most provinces and cities in China still have overcapacity.

#### Environmental regulation (ER)

This article uses comprehensive indicators to measure the intensity of environmental regulations. Three indicators: the ratio of the operating cost of waste gas treatment facilities to the industrial output value, the ratio of the operating cost of wastewater treatment facilities to the industrial output value, and the comprehensive utilization rate of solid waste are used to calculate the intensity of environmental regulations^35^.

The calculation method for the intensity of environmental regulations is as follows:

First, standardize each indicator to reduce the influence of extreme values and dimensional differences; then take the average value of the standardized indicators. The specific calculation process is as follows:

19 $$\begin{array}{c}h{g}_{ij}^{w}=\left[h{g}_{ij}-\mathrm{min}\left(h{g}_{j}\right)\right]/\left[\mathrm{max}\left(h{g}_{j}\right)-\mathrm{min}\left(h{g}_{j}\right)\right]\end{array}$$

Among them, $h{g}_{ij}^{w}$ is the standardized value of category $j$ indicators in the $i$ area, $h{g}_{ij}$ represents the original value of category $j$ indicators; $\mathrm{min}\left(h{g}_{j}\right)$ is the minimum value of category $j$ indicators, and $\mathrm{max}(h{g}_{j})$ is the maximum value of category $j$ indicators.

20 $$\begin{array}{c}er=\sum_{j=1}^{3}h{g}_{ij}^{w} /3\end{array}$$

#### Control variables

To control the impact of other variables on the high-quality development of the manufacturing industry, this paper selects profitability, foreign investment, and government participation as the control variables.

Profitability (PS): Manufacturing companies have a good level of profitability, which is the key to the sustainable development and stable operation of the manufacturing industry. This article takes the total profit of industrial enterprises above the designated size as the proxy variable of the profitability of the manufacturing industry^36^.

Foreign investment (FDI) Foreign investment can not only promote economic growth but also bring about technological spillovers, thereby accelerating technological progress^13^. FDI is mainly concentrated in the high- and mid-end technology industries of the manufacturing industry. The development quality of high-end technology manufacturing is the key to the high-quality development of the manufacturing industry. Expressed by the amount of foreign investment utilized by each province.

#### The degree of government participation (GP)

The development of the manufacturing industry is inseparable from the government's financial support. The strategic layout formulated by the local government for the development of the manufacturing industry is the key to the development of the manufacturing industry, and fiscal expenditures have a driving effect on the development of the manufacturing industry. The degree of government participation (GP) is expressed by the ratio of local government fiscal expenditure to GDP^37^.

The descriptive statistics of each variable are shown in Table 2.

**Table 2:** Descriptive statistics of variables.

Variable	Mean value	Standard deviation	Maximum value	Minimum value
Quality	0.54	0.14	0.94	0.19
CU	0.80	0.18	1	0.36
ER	0.45	0.18	0.96	0.03
PS	1532.35	1764.26	9686.84	0.01
FDI	606,686.30	705,400.70	3,575,956	2044
GP	0.21	0.09	0.63	0.08

### Data sources

Because of the consistency of the unified caliber and the basic data (due to lack of data in Tibet and Taiwan), this article uses panel data from 30 provinces and cities in China from 2005 to 2015, data from China Statistical Yearbook, "China Industrial Statistical Yearbook", "China Statistical Yearbook"," China Industrial Economic Statistical Yearbook", "China Environmental Statistical Yearbook"," China Science and Technology Statistical Yearbook", WIND Database.

## Metrology economics model construction

### Panel regression model construction

The panel regression model constructed in this paper is as follows:

21 $$\begin{array}{c}{Quality}_{it}=c+{\beta }_{1}C{U}_{it}+{\beta }_{2}E{R}_{it}+{\beta }_{3}G{P}_{it}+{\beta }_{4}lnFD{I}_{it}+{\beta }_{5}lnP{S}_{it}+{\varepsilon }_{it}\end{array}$$

The *Quality* is the interpretation variable, which represents the high-quality development of the manufacturing industry; CU is the core interpretation variable, which represents capacity utilization; ER is environmental regulation, GP, FDI, and PS are control variables, representing the degree of government participation, foreign investment, Profit status. $i$ means each province; $t$ represent the year; $\varepsilon$ is a random variable, and ${\beta }_{1}-{\beta }_{4}$ is the coefficient of each variable.

Note: Because of these variables, the four indicators: Quality, ER, CU, and GP are all calculated rates of change, not horizontal quantities. Therefore, the logarithm of these four indicators is not taken in the regression model.

### Threshold regression model construction

To further explore the impact of the capacity utilization rate on the manufacturing industry at different environmental regulation strengths, with the environmental regulation (ER) as the threshold variable, based on the data existing herein, this article establishes a single threshold regression model and the double threshold regression model as follows:

22 $$\begin{array}{c}{Quality}_{it}=C+{\beta }_{1}{CU}_{it}I\left({ER}_{it}\le \gamma \right)+{\beta }_{2}{CU}_{it}I\left({ER}_{it}>\gamma \right)+{\alpha }_{1}{GP}_{it}+{\alpha }_{2}ln{FDI}_{it}+{\alpha }_{3}ln{PS}_{it}+{\mu }_{i}+{\varepsilon }_{it}\end{array}$$

23 $$\begin{array}{c}{Quality}_{it}=C+{\beta }_{1}{CU}_{it}I\left(E{R}_{it}\le {\gamma }_{1}\right)+{\beta }_{2}{CU}_{it}I\left({\gamma }_{1}<E{R}_{it}\le {\gamma }_{2}\right)+{\beta }_{3}C{U}_{it}I\left({\gamma }_{2}<E{R}_{it}\right)\\ +{\alpha }_{1}{GP}_{it}+{\alpha }_{2}ln{FDI}_{it}+{\alpha }_{3}ln{PS}_{it}+{\mu }_{i}+{\varepsilon }_{it}\#\end{array}$$

In the model, I represent an indication function (when the condition is satisfied, its value is 1, otherwise it is 0), ${Quality}_{it}$ is the dependent variable, representing the high-quality development of the manufacturing industry; ${CU}_{it}$ is the independent variable, representing the capacity utilization rate; ${ER}_{it}$ is the threshold variable, representing the intensity of environmental regulations; ${GP}_{it}$, ${FDI}_{it}$, ${PS}_{it}$ are control variables, indicating the profit status, foreign investment, and government participation; ${\mu }_{i}$ is an individual effect of each region; ${\varepsilon }_{it}$ is a random error.

## Empirical results and analysis

### Panel data analysis results

This article uses mixed regression, fixed effects, and random effects to process the data. For fixed effect, F statistic is to test all the individual effect overall significance, F statistic probability is 0.0000, the test results show that the fixed effect model is better than mixed OLS model. For random effects, the LM test obtains the value of P 0.0000, indicating that the random effect is very significant, and the random effect model is also superior to the mixed OLS model. As a comparison, the MLE estimation of the random effect model, the LR test p-value is 0.0000, and the individual random effect is considered to exist, and the random effect is also proved to be superior to mixed regression. In the selection of random effect model and fixed-effect model, Hausman tested p-value > 0.05 and thought that random effect model RE is better than fixed-effect model FE, so this article uses random effect model RE estimation results for analysis. The results are shown in Table 3.

**Table 3:** Panel regression results.

The explanatory variables	OLS	MLE	FE	RE
CU	0.1583 (0.1044)	0.1711* (0.0695)	0.1807* (0.1078)	0.1723** (0.0728)
ER	− 0.0736 (0.0496)	− 0.0744* (0.0326)	− 0.0624* (0.0335)	− 0.0740** (0.0328)
Lnps	0.0107 (0.0064)	0.0124 (0.0063)	0.0138** (0.0067)	0.0125* (0.0063)
lnfdi	0.0215*** (0.0081)	0.0266** (0.0083)	0.0564*** (0.0127)	0.0276** (0.0084)
gp	0.6295*** (0.1447)	0.5410*** (0.1131)	0.1186 (0.1755)	0.5270*** (0.1137)
_cons	− 0.0274 (0.1099)	− 0.0943 (0.1032)	− 0.3963** (0.1471)	− 0.1048 (0.1046)
Hausman			Prob > chi^2^ = 0.1375	
R^2^	0.1888		0.2211	0.2017

***, ** and * respectively indicate that the parameter estimation is significant at the levels of 0.01, 0.05, and 0.1.

According to the random effect RE estimate, capacity utilization has a significant positive effect on the high-quality development of the manufacturing industry, with a coefficient of action of 0.1723 (0.0728) and a 5% test, hypothesis H1 is proven. This shows that the improvement of production capacity utilization in various regions has promoted the development speed, development benefits, and structural optimization of the manufacturing industry, and promoted the high-quality development of manufacturing. Environmental regulation has a significant negative effect on the high-quality development of the manufacturing industry, with a coefficient of − 0.0740 (0.0328) and passed the 5% test; This shows that strict environmental regulations have aggravated the company's high energy consumption, high pollution, and negative impact on the production capacity of manufacturing high-quality development. The level of profitability, foreign direct investment, and government participation all have significant positive effects on the high-quality development of the manufacturing industry. (Passed 10%, 5%, 1% tests, respectively). This shows that the manufacturing industry has a good level of profitability is conducive to the high-quality development of the manufacturing industry, the expansion of FDI can be called an important economic means of manufacturing development, The advanced equipment and management concepts that accompany FDI promote the development of manufacturing industry, and the participation of local governments is conducive to the integration of resources of manufacturing enterprises and the high-quality development of manufacturing industry.

### Threshold regression result analysis

In this article, the nonlinear relationship between the high-quality development of the manufacturing industry is empirically tested under the condition that environmental regulation is the threshold variable. Based on Hansen's threshold effect regression model estimation method, the threshold estimates and related parameters are obtained^38^. This mainly includes the use of Bootstrap (self-sampling) method to construct F statistics and P values to test the significance of threshold effect; Using LR (seemingly proportional) statistics to verify the veracity of whether the threshold estimate is equal to the true value, Hansen estimated the confidence interval of LR(q). In cases where the significance level is $\alpha$, the original hypothesis cannot be rejected when $LR\left(q\right)\le -2\mathrm{ln}(1-\sqrt{1-\alpha })$. In general, the statistical threshold for LR is 7.3523 at the $\alpha$ at a significance level of 5%. The single threshold and double threshold are tested respectively to determine the number of threshold values and model types. The threshold effect test results are shown in the Table 4. The single threshold effect passed the 5% significance test, and the double threshold effect was not significant. Therefore, in the model of high-quality development effect of capacity utilization on the manufacturing industry, there is a single threshold value of environmental regulation, and the threshold estimate is 0.6727. The threshold estimate and confidence interval are shown in Table 5.

**Table 4:** Threshold effect test results.

Threshold inspection	F statistics	P-value	Bootstrap times	1% threshold	5% threshold	10% threshold
A single threshold	14.66**	0.030	300	10.967	13.677	16.460
Double threshold	6.64	0.257	300	9.774	12.273	22.841

**indicate that the parameter estimation is significant at the levels of 0.01, 0.05, and 0.1.

**Table 5:** Threshold estimate and confidence interval.

Threshold inspection	Threshold estimate	95% confidence interval
A single threshold	0.6727	[0.6229,0.6730]

The estimation results of threshold regression model parameters are shown in Table 6, for the core explanatory variable capacity utilization rate and threshold variable environmental regulation, when the environmental regulation is less than the threshold estimate (0.6727), the capacity utilization rate has a significant positive impact on the high-quality development of the manufacturing industry, the coefficient of action is 0.2248 (0.031); When the environmental regulation is greater than the threshold value (0.6727), the capacity utilization rate has a non-significant positive effect on the high-quality development of the manufacturing industry, and the coefficient of action is 0.1416 (0.183), hypothesis H2 is proven.

**Table 6:** Threshold model coefficient and its test.

Variables	Coefficient	T-value	P-value
GP	0.0123**	2.05	0.041
FDI	0.0574***	4.60	0.000
PS	0.1249	0.72	0.470
$ER\le$ 0.6727	0.2248**	2.17	0.031
*ER*$>$ 0.6727	0.1416	1.34	0.183
Cons	− 0.4627***	− 3.41	0.001
R_squared		0.2466	

*** and ** respectively indicate that the parameter estimation is significant at the levels of 0.01, 0.05, and 0.1.

For the control variables, in the threshold regression model, the profit situation has a significant positive influence on the high-quality development of the manufacturing industry, and the coefficient of action is 0.0123 (0.041); Foreign direct investment has a significant positive effect on the high-quality development of manufacturing industry, with a factor of 0.0574 (0.000) and a positive effect of government participation on the high-quality development of manufacturing industry, with a factor of action of 0.1249 (0.470).

**Figure 4:**
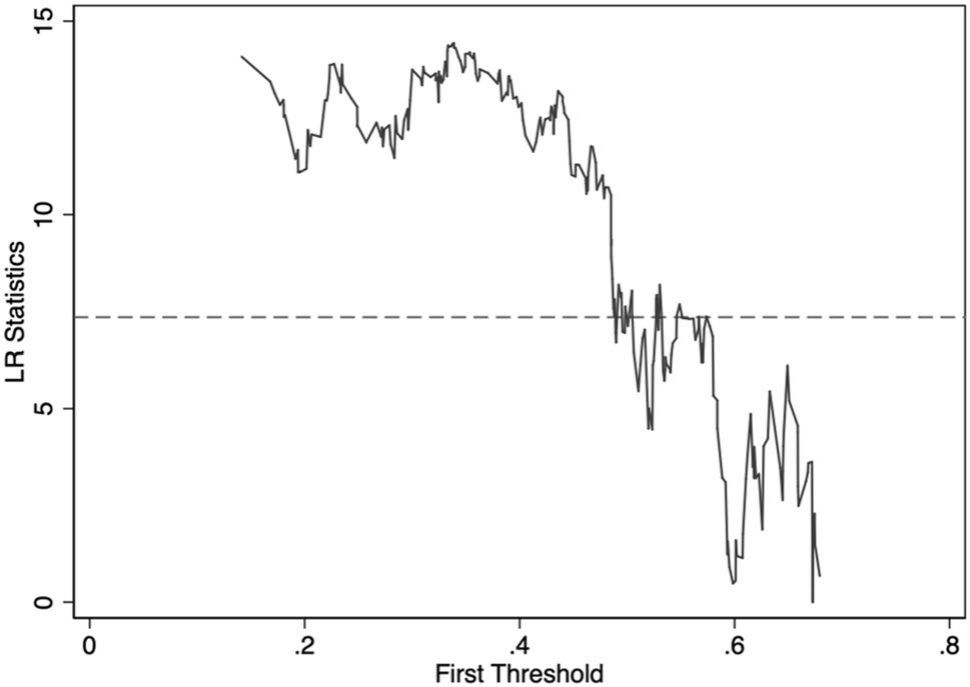
Likelihood ratio function graph of threshold 0.6727.

To verify the authenticity of the threshold estimation, the following uses the LR (likelihood ratio) statistic to the consistency of the dual-threshold estimates to the real value. After the threshold effect test, to more clearly and intuitively understand the double threshold estimation and confidence interval, the LR function diagram is used to test the authenticity and confidence interval of the double threshold estimation, as shown in Fig. 4.

Due to the obvious differences in environmental governance and manufacturing development in various regions of China, the single threshold effect of environmental regulation is analyzed to analyze the distribution between different regions. Table 7 shows that in 2005-2015, the number of areas with high environmental regulations in china's provinces and cities increased first and then decreased. Most of China's provinces and cities over the years in the low environmental regulation intensity range (ER ≤ 0.6727), 2006–2010 in the high environmental regulation intensity (ER > 0.6727) significantly increased. In 2007 and 2009, the largest number of areas were in high environmental regulation, accounting for 29% (30 provinces and municipalities). From the perspective of regional distribution, the proportion of the western region is the largest, and the proportion of the central region is in high environmental regulations.

**Table 7:** The distribution of provinces and cities under different threshold intervals over the years.

Year	*ER* ≤ 0.6.727	*ER* > 0.6727
2005	Beijing, Tianjin, Hebei, Shanxi, Inner Mongolia, Liaoning, Jilin, Heilongjiang, Shanghai, Jiangsu, Zhejiang, Anhui, Fujian, Jiangxi, Shandong, Henan, Hubei, Hunan, Guangdong, Hainan, Chongqing, Sichuan, Guizhou, Yunnan, Shaanxi, Gansu, Qinghai, Ningxia, Xinjiang	Guangxi
2006	Beijing, Tianjin, Hebei, Shanxi, Inner Mongolia, Heilongjiang, Shanghai, Jiangsu, Anhui, Fujian, Jiangxi, Henan, Hubei, Hunan, Guangxi, Hainan, Chongqing, Guizhou, Yunnan, Shaanxi, Gansu, Qinghai, Ningxia	Liaoning, Jilin, Zhejiang, Shandong, Guangdong, Sichuan, Xinjiang
2007	Tianjin, Shanxi, Jilin, Heilongjiang, Shanghai, Jiangsu, Zhejiang, Anhui, Fujian, Jiangxi, Henan, Hubei, Hainan, Chongqing, Sichuan, Guizhou, Yunnan, Shaanxi, Gansu, Qinghai, Ningxia	Beijing, Hebei, Inner Mongolia, Liaoning, Shandong, Hunan, Guangdong, Guangxi, Xinjiang
2008	Beijing, Tianjin, Hebei, Shanxi, Inner Mongolia, Liaoning, Jilin, Heilongjiang, Shanghai, Jiangsu, Zhejiang, Anhui, Fujian, Jiangxi, Shandong, Hubei, Hunan, Sichuan, Guizhou, Shaanxi, Qinghai, Ningxia, Xinjiang	Henan, Guangdong, Guangxi, Hainan, Chongqing, Yunnan, Gansu
2009	Beijing, Tianjin, Shanxi, Inner Mongolia, Liaoning, Jilin, Heilongjiang, Shanghai, Jiangsu, Zhejiang, Anhui, Fujian, Henan, Hubei, Hunan, Hainan, Chongqing, Guizhou, Gansu, Qinghai, Xinjiang	Hebei, Jiangxi, Shandong, Guangdong, Guangxi, Sichuan, Yunnan, Shaanxi, Ningxia
2010	Beijing, Tianjin, Hebei, Shanxi, Inner Mongolia, Liaoning, Jilin, Heilongjiang, Shanghai, Jiangsu, Zhejiang, Anhui, Fujian, Henan, Hubei, Hunan, Guangxi, Hainan, Chongqing, Shaanxi, Gansu, Xinjiang	Jiangxi, Shandong, Guangdong, Sichuan, Guizhou, Yunnan, Qinghai, Ningxia
2011	Beijing, Hebei, Shanxi, Inner Mongolia, Liaoning, Jilin, Heilongjiang, Jiangsu, Zhejiang, Anhui, Fujian, Jiangxi, Henan, Hunan, Guangdong, Guangxi, Hainan, Chongqing, Sichuan, Yunnan, Shaanxi, Gansu, Qinghai, Ningxia, Xinjiang	Tianjin, Shanghai, Shandong, Hubei, Guizhou
2012	Beijing, Tianjin, Hebei, Shanxi, Inner Mongolia, Liaoning, Jilin, Heilongjiang, Jiangsu, Zhejiang, Anhui, Fujian, Jiangxi, Shandong, Henan, Hubei, Hunan, Guangdong, Guangxi, Hainan, Chongqing, Sichuan, Yunnan, Shaanxi, Gansu, Ningxia, Xinjiang	Shanghai, Guizhou, Qinghai
2013	Beijing, Tianjin, Hebei, Shanxi, Inner Mongolia, Liaoning, Jilin, Heilongjiang, Shanghai, Jiangsu, Zhejiang, Anhui, Jiangxi, Shandong, Henan, Hubei, Hunan, Guangdong, Guangxi, Hainan, Chongqing, Sichuan, Guizhou, Yunnan, Shaanxi, Gansu, Qinghai, Ningxia, Xinjiang	Fujian
2014	Beijing, Tianjin, Hebei, Shanxi, Inner Mongolia, Liaoning, Jilin, Heilongjiang, Shanghai, Jiangsu, Zhejiang, Anhui, Fujian, Jiangxi, Shandong, Henan, Hubei, Hunan, Guangdong, Guangxi, Hainan, Chongqing, Sichuan, Guizhou, Shaanxi, Gansu, Qinghai, Xinjiang	Yunnan, Ningxia
2015	Beijing, Tianjin, Shanxi, Inner Mongolia, Liaoning, Jilin, Heilongjiang, Shanghai, Jiangsu, Fujian, Jiangxi, Shandong, Henan, Hubei, Hunan, Guangdong, Guangxi, Hainan, Chongqing, Sichuan, Guizhou, Yunnan, Gansu, Qinghai, Ningxia, Xinjiang	Hebei, Zhejiang, Anhui, Shaanxi

## Conclusions and practices

Based on the panel data of 30 provinces and cities in China from 2005 to 2015, this article analyzes the impact of capacity utilization on the high-quality development of the manufacturing and establishes a nonlinear threshold regression model on this basis, and studies and analyzes environmental regulations as a threshold variable Under the influence of capacity utilization rate on the high-quality development of the manufacturing industry. The research results show that: capacity utilization, profitability, foreign direct investment, and government participation all have a significant positive impact on the high-quality development of the manufacturing industry; environmental regulations have a significant negative impact on the high-quality development of the manufacturing industry. And in the model of the effect of capacity utilization on the high-quality development of the manufacturing industry, environmental regulation has a single threshold effect. With the increase in the intensity of environmental regulation, the coefficient and significance of the effect of capacity utilization on the high-quality development of the manufacturing industry have changed. From a significant positive impact to an insignificant positive impact, and the coefficient of action gradually decreases.

Based on the above empirical research conclusions, combined with the development status of China's manufacturing industry, the management revelation and countermeasures for promoting high-quality development of manufacturing industry are proposed as follows: (1) The production capacity utilization has a remarkable positive impact on the high-quality development of manufacturing, and overcapacity will hinder the high-quality development of manufacturing. In recent years, the excess capacity of my China’s has been improved, and some areas also need to combine the background of high-quality development and use strong means to relieve overcapacity. (2) Establishing the environmental regulation intensity of China's national conditions, optimizing environmental protection and capacity governance, properly strengthening environmental governance in China, maximizing the role of stimulating environmental regulation in the manufacturing industry. (3) The manufacturing enterprise has a good profit level is the key to the sustainable development of the manufacturing industry, and the manufacturing enterprise should formulate the correct strategic plan to adjust the management model to ensure the quality of business and profitability. (4) Optimize the FDI structure, formulate relevant policies, guide and encourage FDI investment, introduce foreign advanced equipment technology, patents, etc., encourage foreign-invested to help to manufacture high-quality developments such as information technology. (5) Government participates in the integration of manufacturing integration resources in various regions, and the Government should support manufacturing innovation and support the investment of traditional enterprise research funds, and support the new model of the manufacturing industry.

## Limitation and further research

This paper only studies the relationship between capacity utilization, environmental regulation and high-quality development of manufacturing industry. There are many related factors that will have an impact on high-quality development of manufacturing industry, which have not been fully considered. Other factors will be added to analyze in future research. This paper is only based on the Chinese background. Environmental issues are also the focus of research in many countries, and will be further improved in the future research.
